# Risk and Prognosis of Secondary Rectal Cancer After Radiation Therapy for Pelvic Cancer

**DOI:** 10.3389/fonc.2020.584072

**Published:** 2020-10-29

**Authors:** Runkun Yang, Xu Guan, Enrui Liu, Ran Wei, Zhixun Zhao, Haipeng Chen, Zheng Liu, Ming Yang, Zheng Jiang, Xishan Wang

**Affiliations:** ^1^ Department of Colorectal Surgery, The Second Affiliated Hospital of Harbin Medical University, Harbin, China; ^2^ Department of Colorectal Surgery, National Cancer Center/Cancer Hospital/National Clinical Research Center for Cancer, Chinese Academy of Medical Sciences and Peking Union Medical College, Beijing, China; ^3^ Colorectal Cancer Institute, Harbin Medical University, Harbin, China

**Keywords:** pelvic cancer, radiation therapy, second primary cancer, rectal cancer, Surveillance; Epidemiology; End Results database

## Abstract

**Background:**

The relationship between pelvic radiation therapy (RT) and second primary rectal cancer (SPRC) is unclear. The aim of this study was to assess the risk and prognosis of SPRC after pelvic RT.

**Materials and Methods:**

Data for patients who had primary pelvic cancer (PPC) between 1973 and 2016 were retrieved from the Surveillance, Epidemiology, and End Results (SEER) database. Multiple primary standardized incidence ratios (SIRs) were used to assess the risk of SPRC. Five-year overall survival (OS) and rectal cancer-specific survival (RCSS) were calculated using Kaplan–Meier curves.

**Results:**

A total of 573,306 PPC patients were included, 141,225 of whom had been treated with RT. Primary cancers were located in the prostate (50.83%), bladder (24.18%), corpus uterus (16.26%), cervix (5.83%), and ovary (2.91%). A total of 1,491 patients developed SPRC. Overall, the patients who received RT were at increased risk of developing SPRC (SIR = 1.39, 95% confidence interval [CI]: 1.27–1.52). The risk of SPRC decreased in patients who did not undergo RT (SIR = 0.85, 95% CI: 0.80–0.91). The SIR for SPRC in patients who underwent external beam radiation therapy (EBRT) was 1.22 (95% CI: 1.09–1.36). The SIR for SPRC in patients who underwent a combination of EBRT and brachytherapy (EBRT–BRT) was 1.85 (95% CI: 1.60–2.14). For patients who received RT, the SIR for SPRC increased with time after a 5-year latency period from PPC diagnosis. The survival of RT-treated SPRC patients was significantly worse than that of patients with primary rectal cancer only (PRCO).

**Conclusions:**

Patients receiving pelvic RT were at an increased risk of developing SPRC. Different pelvic RT treatment modalities had different effects on the risk of SPRC. We suggest that long-term surveillance of SPRC risk is required for patients who have undergone pelvic RT, especially young patients.

## Introduction

Radiation therapy (RT) has formed part of the treatment regimen for at least 50% of all cancer patients because of the associated reduction in recurrence and improved prognosis (1, 2). RT destroys cancer cells by depositing high-energy radiation on cancer tissues. High doses of ionizing radiation can directly or indirectly (through the production of free radicals) damage the genome of the cell (3). Nevertheless, acute and late toxicity due to RT cannot be ignored.

The risk of developing a second primary cancer (SPC) is one form of late toxicity (4). Nearly 1 in 12 patients diagnosed with a common cancer develop a SPC. More than 55% of patients with two incident cancers die as a result of a secondary cancer (5). During RT for pelvic cancer, the rectum is likely to receive more radiation than organs in the non-pelvic area (6–8), and it is important to understand how radiation affects SPC risk within the field of irradiation.

Studies have shown conflicting results for second primary rectal cancer (SPRC) risk after pelvic RT (9–11). A study based on a Dutch population demonstrated that patients receiving pelvic RT were at an increased risk of developing a SPRC (12), while other studies reported that the tumor incidence in patients receiving pelvic RT did not differ from that of the general population (13, 14). These observations indicate that the relationship between pelvic RT and SPRC requires further determination. Theoretically, different RT modalities may have different effects on SPRC risk; however, there is currently insufficient evidence to reach such a conclusion (15–17).

Compared with primary rectal cancer (PRC), the etiology of RT-related SPRC can be very different. Moreover, whether SPRCs and PRCs are equally sensitive to RT after pelvic RT treatment remains unclear (18). In addition, fibrosis resulting from pelvic RT might make complicate surgery and lead to an increase in surgery-related complications (19). Because of these differences, the survival outcomes between PRC and RT-related SPRC patients might be different.

The aims of the present study were to identify how pelvic RT affects the risk of developing SPRC and compare the survival outcome between PRC and RT-related SPRC patients.

## Methods

### Data Source

The processed, publicly available data on the SEER database was access between January 1, 1973, and December 31, 2016, from 9 registries (Atlanta, Connecticut, Detroit, Hawaii, Iowa, New Mexico, San Francisco–Oakland, Seattle–Puget Sound, and Utah). The demographic and incidence data collected by the SEER registries cover approximately 28% of the US population, and are considered representative of the US population as a whole. Because patients’ records in the SEER database were anonymized and de-identified before analysis, information on cancer cases can be retrieved from the database. The study design was approved by the Ethics Committee of the National Cancer Center/National Clinical Research Center for Cancer/Cancer Hospital; the Chinese Academy of Medical Sciences and Peking Union Medical College institutional review board; and the Second Affiliated Hospital of Harbin Medical University review board. All the methods performed in our study followed the latest guidelines stated in the SEER database.

### Identification of First Primary Pelvic Cancer (PPC)

Solid pelvic cancers in five sites that are routinely treated with RT were included as first primary cancers in this cohort study, including cancers of the cervix uteri, corpus and uterus, ovary, prostate, and urinary bladder. The SEER database strictly adheres to the coding rules for the classification of topography or histology of the International Classification of Diseases for Oncology (ICD-O) guidelines to identify multiple primary malignancies and distinguish between primary and recurrent cancers. The exclusion criteria for a first pelvic primary cancer in this study were as follows: patients with distant cancers; patients under 20 years of age; and patients of unknown race, survival month, cause of death, or RT status.

### Treatment for PPC

The SEER database contains information on the first course of treatment. In this study, patients with a first PPC were classified according to the type of RT received, including external beam radiation therapy (EBRT), a combination of EBRT with brachytherapy involving implants or isotopes (EBRT–BRT), and no radiation therapy (NRT). However, the dosages of radiation administered were not registered in the SEER database.

### Identification of SPRC

Because it takes 5 years from radiation exposure to solid cancer induction (20), the primary outcome of interest was the development of SPRC or second primary rectosigmoid cancer, which was defined as a nonsynchronous malignancy occurring within 5 years after treatment of the first PPC. In addition, patients diagnosed with a third-order or higher multiple PRC were excluded from the study.

### Identification of Primary Rectal Cancer Only (PRCO)

Patients with PRCO were defined as those presenting only with PRC, and without any other malignancy diagnosed during their lifetime.

### Statistical Analysis

Baseline patient and tumor characteristics were compared using the *χ* ^2^ test or Fisher’s exact test, in case of an expected cell count <5. Survival outcomes were calculated using Kaplan–Meier curves, and the log-rank test was applied to compare these curves. The definition of overall survival (OS) was the time from SPRC diagnosis to the date of all-cause death, and the definition of rectal cancer-specific survival (RCSS) was the time from SPRC diagnosis to the date of SPRC-cause death.

SPSS (version 22.0; IBM) was used for the analysis of characteristics and survival, and a *p*-value <0.05 was considered statistically significant. Multiple primary standardized incidence ratios (SIRs) were used as a key measure of the risk of developing a SPC. Here, SIR was defined as the ratio of SPRC incidence to the number of expected SPRC cases in the general US population according to the SEER ascertainment area. The SIR results were stratified by gender, age, and calendar time, and a *p*-value <0.05 (two-sided) was considered statistically significant. Exact Poisson methods were used to calculate 95% confidence intervals (CIs) for the ratio of observed events to expected events (21). All SIR analyses were conducted using SEER*Stat software, version 8.3.6.d.

## Results

### Patient Characteristics

We identified 573,306 patients meeting the selection criteria (average age, 65 years). Patient demographics are depicted in **Table 1**. A total of 141,225 patients (24.63%) were treated with RT for a PPC, 103,947 of which were treated with EBRT only, and 37,278 with EBRT–BRT. Meanwhile, 432,081 (75.37%) patients received no RT. Primary cancers were located in the prostate (50.83%), bladder (24.18%), corpus uteri (16.26%), cervix (5.83%), and ovary (2.91%).

**Table 1:** Characteristics of the primary pelvic cancer (PPC) patients.

	Total *N* = 574,253		NRT *N* = 432,422		RT *N* = 141,831		RT Type			
							EBRT *n* = 104,194		EBRT–BRT *n* = 37,637	
	*n* ^a^	*n* (%) or mean ± SD	*n* ^a^	*n* (%) or mean ± SD	*n* ^a^	*n* (%) or mean ± SD	*n* ^a^	*n* (%) or mean ± SD	*n* ^a^	*n* (%) or mean ± SD
**Age at diagnosis**	573,306	65.00 ± 12.36	432,081	64.67 ± 12.75	141,225	66.02 ± 11.04	103,947	66.02 ± 11.04	37,278	62.02 ± 12.11
≤60		191,026 (33.32)		152,976 (35.40)		38,050 (26.94)		23,165 (22.29)		14,885 (39.93)
>60		382,280 (66.68)		279,105 (64.60)		103,175 (73.06)		80,782 (77.71)		22,393 (60.07)
**Gender**	573,306		432,081		141,225		103,947		37,278	
Male		395,647 (69.01)		297,433 (68.84)		98,214 (69.54)		79,563 (76.54)		18,651 (50.03)
Female		177,659 (30.99)		134,648 (31.16)		43,011 (30.46)		24,384 (23.46)		18,627 (49.97)
**Race**	573,306		432,081		141,225		103,947		37,278	
White		479,744 (83.68)		369,500 (85.52)		110,244 (78.06)		81,755 (78.65)		28,489 (76.42)
Black		57,981 (10.11)		37,817 (8.75)		20,164 (14.28)		14,296 (13.75)		5,868 (15.74)
Other		35,581 (6.21)		24,764 (5.73)		10,817 (7.66)		7,896 (7.60)		2,921 (7.84)
**SPC**	573,306		432,081		141,225		103,947		37,278	
Yes		91,359 (15.94)		69,253 (16.03)		22,106 (15.65)		16,366 (15.74)		5,740 (15.40)
No		481,947 (84.06)		362,828 (83.97)		119,119 (84.35)		87,581 (84.26)		31,538 (84.60)
**SPRC**	573,306		432,081		141,225		103,947		37,278	
Yes		1,491 (0.26)		979 (0.23)		512 (0.36)		325 (0.31)		187 (0.50)
No		571,815 (99.74)		431,102 (99.77)		140,713 (99.64)		103,622 (99.69)		37,091 (99.50)
**Site**	573,306		432,081		141,225		103,947		37,278	
Cervix		33,428 (5.83)		17,003 (3.94)		16,425 (11.63)		5,930 (5.70)		10,495 (28.15)
Corpus uteri		93,201 (16.26)		69,980 (16.20)		23,221 (16.44)		15,128 (14.55)		8,093 (21.71)
Ovary		16,680 (2.91)		15,861 (3.67)		819 (0.58)		795 (0.76)		24 (0.06)
Prostate		291,395 (50.83)		199,655 (46.21)		91,740 (64.96)		73,106 (70.33)		18,634 (49.99)
Bladder		138,602 (24.18)		129,582 (29.99)		9,020 (6.39)		8,988 (8.65)		32 (0.09)
**Grade**	516,100		386,043		130,057		97,798		32,259	
I~II		315,974 (61.22)		249,194 (64.55)		66,780 (51.35)		49,537 (50.65)		17,243 (53.45)
III~IV		200,126 (38.78)		136,849 (35.45)		63,277 (48.65)		48,261 (49.35)		15,016 (46.55)
**Stage**	573,306		432,081		141,225		103,947		37,278	
Localized		221,450 (38.63)		198,864 (46.02)		22,586 (15.99)		14,717 (14.16)		7,869 (21.11)
Regional		60,461 (10.55)		33,562 (7.77)		26,899 (19.05)		16,124 (15.51)		10,775 (28.90)
Localized/regional (prostate-related cases)		291,395 (50.83)		199,655 (46.21)		91,740 (64.96)		73,106 (70.33)		18,634 (49.99)
**Chemotherapy**	573,306		432,081		141,225		103,947		37,278	
Yes		40,210 (7.01)		27,807 (6.44)		12,403 (8.78)		8,000 (7.70)		4,403 (11.81)
No		533,096 (92.99)		404,274 (93.56)		128,822 (91.22)		95,947 (92.30)		32,875 (88.19)
**Surgery**	569,351		429,758		139,593		103,154		36,439	
Yes		393,432 (69.10)		346,820 (80.70)		46,612 (33.39)		35,393 (34.31)		11,219 (30.79)
No		175,919 (30.90)		82,938 (19.30)		92,981 (66.61)		67,761 (65.69)		25,220 (69.21)

a Number of patients for whom data was available.

NRT, no radiation therapy; RT, radiation therapy; EBRT, external beam radiation therapy; EBRT–BRT, combination of external beam with brachytherapy; SPC, second primary cancer; SPRC, second primary rectal cancer.

After a minimum latency of five years from PPC diagnosis, a total of 91,359 patients (15.94%) developed a SPC. Among patients who underwent RT, 15.65% went on to develop a SPC. In total, 16,366 (15.74%) patients in the EBRT group and 5,740 (15.40%) patients in the EBRT–BRT group developed a SPC. In the NRT group, 69,253 (16.03%) patients developed a SPC. The number of patients who developed a SPRC was 1,491 (0.23%), 325 (0.31%), and 187 (0.50%) for the NRT, EBRT, and EBRT–BRT groups, respectively. The above data showed that, compared with the NRT group, a greater proportion of patients who received EBRT and EBRT–BRT for their PPC developed a SPRC; no difference was found between the latter two groups.

### Comparison of SPRC With and Without RT

The characteristics of the patients who developed a SPRC after a minimum latency of five years from PPC diagnosis are shown in **Table 2**. Patients who developed a SPRC after RT (RT-SPRC) were older than those who developed a SPRC without RT (NRT-SPRC) (76.20 ± 9.20 *vs*. 74.74 ± 10.29, *p* = 0.007). There was no significant difference between the NRT-SPRC group and the RT-SPRC group in terms of gender, race, tumor grade, and whether the patients underwent surgery or not. The proportion of SPRCs that were located in the rectum in the RT-SPRC group was significantly higher than that in the NRT-SPRC group (81.25 *vs*. 72.93%, *p* < 0.001). Patients in the RT- SPRC group had a greater proportion of mucinous adenocarcinomas (8.59%) than those in the NRT-SPRC group (4.80%) (*p* = 0.001). Compared with those in the NRT-SPRC group, patients in the RT-SPRC group had a smaller proportion of localized stages (50.75 *vs*. 48.13%, respectively; *p* = 0.045) and a greater proportion of regional stages (33.41 *vs*. 36.68%, respectively; *p* = 0.045). A significantly greater percentage of patients in the NRT-SPRC group received chemotherapy for their SPRC compared with those from the RT-SPRC group (32.89 *vs*. 26.17%, respectively; *p* = 0.009). Only 9.75% of the patients in the RT-SPRC group received RT again for their SPRC. However, 30.34% of the patients in the NRT-SPRC group received RT for their SPRC.

**Table 2:** Comparison of the characteristics of second primary rectal cancer (SPRC) patients receiving radiotherapy (RT) or not.

	NRT-SPRC *n* = 979		RT-SPRC *n* = 512		*p*-value
	*n* ^a^	*n* (%) or mean ± SD	*n* ^a^	*n* (%) or mean ± SD	
**Age at diagnosis**	979	74.74 ± 10.29	512	76.20 ± 9.20	0.007
≤60		96 (9.81)		30 (5.86)	
>60		883 (90.19)		482 (94.14)	
**Gender**	979		512		0.836
Male		632 (64.56)		327 (63.87)	
Female		347 (35.44)		185 (36.13)	
**Race**	979		512		0.178
White		837 (85.50)		419 (81.84)	
Black		74 (7.56)		50 (9.77)	
Other		68 (6.95)		43 (8.40)	
**Site**	979		512		<0.001
Rectum		714 (72.93)		416 (81.25)	
Rectosigmoid junction		265 (27.07)		96 (18.75)	
**Pathological type**	979		512		0.001
Adenocarcinoma		831 (84.88)		436 (85.16)	
Mucinous adenocarcinoma		47 (4.80)		44 (8.59)	
Other		101 (10.32)		32 (6.25)	
**Grade**	775		413		0.43
I~II		632 (81.55)		326 (78.93)	
III~IV		143 (18.45)		87 (21.07)	
**Stage**	865		428		0.045
Localized		439 (50.75)		206 (48.13)	
Regional		289 (33.41)		157 (36.68)	
Distant		137 (15.84)		65 (15.19)	
**Tumor size**	394		239		0.017
≤2 cm		89 (22.59)		39 (16.32)	
>2 cm, ≤5		200 (50.76)		138 (57.74)	
>5 cm		105 (26.65)		62 (25.94)	
**Chemotherapy**	979		512		0.009
Yes		322 (32.89)		134 (26.17)	
No		657 (67.11)		378 (73.83)	
**Radiation**	979		512		<0.001
Yes		297 (30.34)		49 (9.57)	
No		682 (69.66)		463 (90.43)	
**Surgery**	967		504		0.151
Yes		735 (76.01)		366 (72.62)	
No		232 (23.99)		138 (27.38)	

a patients with data available.

NRT-SPRC, SPRC without RT; RT-SPRC, SPRC with RT.

### SIR of SPRC

Compared with the general US population, patients who received RT for their PPC were at an increased risk of developing a SPRC (SIR = 1.39, 95% CI: 1.27–1.52). The SIR of SPRC was 1.22 (95% CI: 1.09–1.36) in the EBRT group and 1.85 (95% CI: 1.60–2.14) in the EBRT–BRT group (**Table 3**). The increased risk of developing a SPRC was due to RT, as evidenced by the SIR of 1.39 in the RT group compared with a SIR of 0.85 for the NRT group (95% CI: 0.80–0.91). The SIR of SPRC tended to be higher in patients who underwent EBRT and EBRT–BRT than in patients who had not received RT. This tendency was found for the cervix uteri, corpus and uterus, prostate, and bladder, but not the ovary. Among patients who did not receive RT, those with prostate cancer (SIR = 0.75, 95% CI: 0.67–0.8) and bladder cancer (SIR = 0.84, 95% CI: 0.75–0.94) were at a reduced risk of developing a SPRC.

**Table 3:** Standardized incidence ratio (SIR) of second primary rectal cancer (SPRC).

	NRT			EBRT			EBRT–BRT		
	Observed	SIR	95% CI	Observed	SIR	95% CI	Observed	SIR	95% CI
Total	979	0.85#	0.80–0.91	325	1.22#	1.09–1.36	187	1.85#	1.60–2.14
**Age at diagnosis (years)**									
20–59	82	1.12	0.89–1.40	8	1.21	0.52–2.38	18	3.00#	1.78–4.74
60–74	365	0.78#	0.7–0.87	97	1.23	1.00–1.50	70	1.77#	1.38–2.24
75+	532	0.88#	0.81–0.96	220	1.21#	1.06–1.38	99	1.79#	1.45–2.18
**Gender**									
Male	632	0.80#	0.74–0.87	236	1.15#	1.01–1.31	91	1.63#	1.32–2.01
Female	347	0.97	0.87–1.08	89	1.43#	1.15–1.76	96	2.12#	1.72–2.59
**Race**									
White	837	0.83#	0.78–0.89	267	1.24#	1.10–1.40	152	1.87#	1.59–2.20
Black	74	0.96	0.75–1.21	31	1.02	0.69–1.44	19	1.49	0.90–2.33
Other	68	1.23	0.96–1.56	27	1.29	0.85–1.87	16	2.35#	1.34–3.82
**Latency**									
60–119 months	509	0.88#	0.8–0.96	163	1.04	0.88–1.21	63	1.22	0.94–1.56
120–239 months	385	0.84#	0.76–0.92	135	1.39#	1.16–1.64	95	2.33#	1.89–2.85
240+ months	85	0.81	0.65–1.00	27	2.14#	1.41–3.12	29	3.42#	2.29–4.91
**Year**									
1975–1984	18	0.85	0.51–1.35	2	0.51	0.06–1.84	1	0.50	0.01–2.80
1985–1994	132	0.92	0.77–1.09	28	1.20	0.80–1.74	21	1.68#	1.04–2.57
1995–2004	271	0.92	0.81–1.03	62	1.13	0.87–1.45	35	1.67#	1.16–2.32
2005+	558	0.82#	0.75–0.89	233	1.26#	1.10–1.43	130	1.99#	1.66–2.36
**Site**									
Cervix	49	1.22	0.90–1.62	19	2.94#	1.77–4.60	49	2.44#	1.80–3.22
Corpus uteri	211	1.00	0.87–1.14	65	1.27	0.98–1.62	47	1.87#	1.38–2.49
Ovary	42	1.21	0.87–1.64	2	0.89	0.11–3.22	0	0	0–100.45
Prostate	359	0.75#	0.67–0.83	220	1.13	0.99–1.29	91	1.63#	1.32–2.01
Bladder	318	0.84#	0.75–0.94	19	1.45	0.87–2.26	0	0	0–174.55

SIR, standardized incidence ratios; CI, confidence interval; NRT, no radiation therapy; EBRT, external beam radiation therapy; EBRT–BRT, combination of external beam with brachytherapy.

^#^p < 0.05.

**Figure 1:**
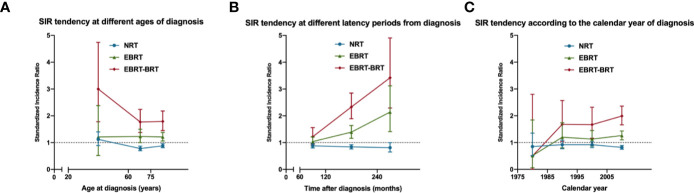
The standardized incidence ratio (SIR) tendency for second primary rectal cancer (SPRC) in surviving pelvic cancer patients. **(A)** SIR tendency at different ages of diagnosis. **(B)** SIR tendency at different latency periods from diagnosis. **(C)** SIR tendency according to the calendar year of diagnosis.

We next calculated the SIRs of SPRC according to age range, gender, race, latency from PPC diagnosis, year of diagnosis, and PPC site, respectively. NRT and EBRT–BRT patients presented a tendency for a deceasing risk of developing SPRC with increasing age at diagnosis (**Figure 1A**). This showed that patients who were younger at PPC diagnosis were at an increased risk of developing SPRC. Furthermore, we found that the risk of developing SPRC increased with time after a 5-year latency from the diagnosis of PPC in the EBRT and EBRT–BRT groups, but not in the NRT group (**Figure 1B**). Additionally, we found that the risk of developing a SPRC increased with increasing calendar year of diagnosis (**Figure 1C**).

### Survival Outcomes for SPRC Patients

We separately matched NRT-SPRC and RT-SPRC patients with PRCO patients by age at diagnosis, gender, race, tumor stage, chemotherapy, radiotherapy, and surgery by propensity score matching (PSM) at a ratio of 1:5. Demographic data of patients after PSM were shown in **Supplementary Data**.

**Figure 2:**
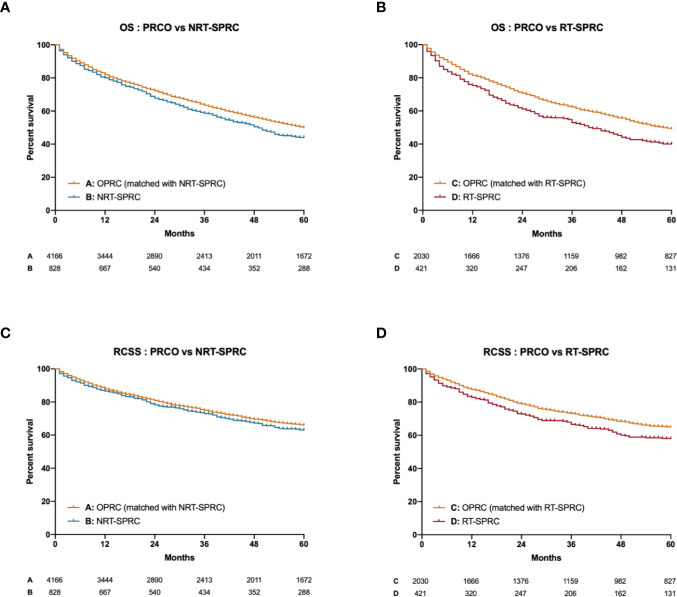
Kaplan–Meier curves for the overall survival (OS) and rectal cancer-specific survival (RCSS) of primary rectal cancer only (PRCO) patients versus those with second primary rectal cancer (SPRC; with radiotherapy [RT] or without radiotherapy [NRT]). **(A)** OS of PRCO versus NRT-SPRC patients. **(B)** OS of PRCO versus RT-SPRC patients. **(C)** RCSS of PRCO versus NRT-SPRC patients. **(D)** RCSS of PRCO versus RT-SPRC patients.

Significant differences in five-year OS were found between NRT-SPRC patients and matched PRCO patients (*p* = 0.002; **Figure 2A**). We also found that the 5-year OS for RT-SPRC patients was significantly shorter than that for matched PRCO patients (*p* < 0.001; **Figure 2B**). The hazard ratio (HR) was 1.18 (95% CI: 1.06–1.31) for the NRT-SPRC group *vs*. the matched PRCO group and 1.33 (95% CI: 1.14–1.55) for the RT-SPRC *vs*. the matched PRCO group. In addition, the survival analysis showed that there was a significant difference in RCSS between RT-SPRC patients and matched PRCO patients (*p* = 0.004, HR = 1.30, 95% CI: 1.07–1.58; **Figure 2D**). No significant difference in RCSS was observed between patients in the NRT-SPRC group and those in the PRCO group (*p* = 0.116; HR = 1.11, 95% CI: 0.97–1.28; **Figure 2C**). These results suggested that the prognosis was worse for RT-receiving SPRC patients than for PRCO patients.

## Discussion

Our SEER-based study was the largest patient cohort in the literature and investigated the relationship between pelvic RT as a treatment for PPC and the risk of developing SPRC. Our data confirmed that patients who received pelvic RT for their PPC were at an increased risk of developing SPRC. Recent studies have reached similar conclusions (4, 16, 21). In addition, our data showed that while a greater proportion of patients who underwent RT also developed SPRC, the proportion of patients who developed SPC after pelvic RT was similar to that for patients who did not receive RT. This indicated that the increased incidence of SPC was associated only with the rectum, which is also the area most likely to be affected by pelvic RT.

Moreover, among patients who received RT for their PPC, the risk of developing a SPRC was higher for patients who received a combination of EBRT and BRT than for those who received EBRT only. Studies have reported that SPC rates were similar overall for high- or low-dose RT treatment for pelvic cancer (risk ratio (RR) = 0.97, 95% CI: 0.89–1.06), as were the rates for site-specific cancers. There was a significant reduction in colon cancer rates following BRT compared with those after EBRT (22). In our study, we did not compare different doses of RT. However, we confirmed that the EBRT–BRT combination increased the SPRC risk compared with EBRT alone, which may have been due to the higher doses of radiation associated with combination treatment. Our results showed that the RT-related SPRC risk was associated with the pelvic RT treatment modality, which may also be associated with the radiation dose. This difference resulting from the different RT modalities employed or different radiation doses cannot be ignored. We suggest that patients undergoing the EBRT–BRT combination treatment should be considered as having a higher SPRC risk.

The risk of SPRC appeared to be reduced among patients who did not receive pelvic RT after a PPC, and a similar observation was made for prostate cancer and bladder cancer. These results were consistent with those of other studies (12, 16).

We also observed that patients diagnosed with a PPC at a younger age were at the highest risk of developing a SPRC. A study based on a Swedish population showed that the SIRs for colorectal cancer were higher for men diagnosed with Hodgkin lymphoma before the age of 35 years than for those diagnosed later in life (23). Clearly, younger patients would have a longer lifespan after being cured, which also implies a greater cancer risk. We also concluded that the risk of developing SPRC increases with time after a 5 year latency period from PPC diagnosis. This tendency was not observed in the NRT group, indicating that the increased risk of developing SPRC was indeed due to the pelvic RT and not other factors. Considering the expanding effect, the latency period of SPRC could be very long indeed. This suggests that a long follow-up is needed for patients undergoing pelvic RT, especially for young patients. Interestingly, we found that the SPRC risk increased with increasing calendar year of diagnosis in RT-treated patients. This tendency did not exist in patients who did not receive RT, indicating that this effect was also due to the RT. With the advancement of RT technology, more cancer patients would have been cured, and an increasing number of pelvic cancer survivors would also result in an increased SPRC potential.

The OS and RCSS were worse for patients in the RT-SPRC group than for those in the PRCO group. For the NRT patients, no significant difference in RCSS was found between them and those in the PRCO group. This indicated that pelvic RT may affect the pathogenesis and biological characteristics of rectal cancer, and lead to differences in survival outcome. Combined with the observation that patients in the RT-SPRC group had a greater incidence of mucinous adenocarcinoma, we suggest that clinicians should consider these differences when treating RT-related SPRC patients and pay special attention to the treatment modality.

This study had several limitations. First, environmental factors that may have had a significant influence on cancer incidence, such as smoking, were not considered as no records were available in the SEER database. Because of the lack of precise RT information in the database, the time frame included in the study does not account for the change to favoring IMRT in many pelvic malignancies. The purpose of this study was to analyze the impact of pelvic RT on SPRC; however, no specific radiation doses were available as a reference, which made it difficult to analyze whether this risk was related to the radiation dose. Nonetheless, as a remedy, we distinguished between patients who underwent EBRT as the only form of RT and those who received a combination of EBRT and BRT. In theory, the EBRT–BRT group should have received higher radiation doses, allowing us to analyze the effect of different RT modalities and doses on the risk of developing SPRC. Also, this study is greatly limited by the number of confounders that might be minimized in a Metanalysis from prospective clinical trials.

In conclusion, compared with the U.S. general population, patients who received RT for PPC were at an increased risk of developing a SPRC. Moreover, this RT-related risk for SPRC was associated with the RT treatment modality they had received. We suggest that a long follow-up time is needed for patients undergoing pelvic RT, especially for young patients. Special consideration should be given to SPRC patients given the differences between this group and PRCO patients.

## Data Availability Statement

The raw data supporting the conclusions of this article will be made available by the authors, without undue reservation.

## Ethics Statement

The studies involving human participants were reviewed and approved by Ethics Committee of National Cancer Center/National Clinical Research Center for Cancer/Cancer Hospital. Written informed consent for participation was not required for this study in accordance with the national legislation and the institutional requirements.

## Author Contributions

XW: writing—review and editing and supervision. XG: conceptualization, investigation, and supervision. RY: conceptualization, investigation, and writing—original draft. EL: software and formal analysis. RW: validation and data curation. ZZ: resources. HC: validation. ZL: investigation and project administration. MY: data curation and supervision. ZJ: data curation and revision. All authors contributed to the article and approved the submitted version.

## Conflict of Interest

The authors declare that the research was conducted in the absence of any commercial or financial relationships that could be construed as a potential conflict of interest.

## Supplementary Material

The Supplementary Material for this article can be found online at: https://www.frontiersin.org/articles/10.3389/fonc.2020.584072/full#supplementary-material

## References

[1] Tabaja L Sidani SM Management of Radiation Proctitis. Dig Dis Sci (2018) 63:2180–8. 10.1007/s10620-018-5163-8 29948565

[2] van Gijn W Marijnen CAM Nagtegaal ID Kranenbarg EM-K Putter H Wiggers T Preoperative radiotherapy combined with total mesorectal excision for resectable rectal cancer: 12-year follow-up of the multicentre, randomised controlled TME trial. Lancet Oncol (2011) 12:575–82. 10.1016/S1470-2045(11)70097-3 21596621

[3] Baskar R Dai J Wenlong N Yeo R Yeoh KW Biological response of cancer cells to radiation treatment. Front Mol Biosci (2014) 1:24. 10.3389/fmolb.2014.00024 25988165 PMC4429645

[4] de Gonzalez AB Curtis RE Kry SF Gilbert E Lamart S Berg CD Proportion of Second Cancers Attributable to Radiotherapy Treatment in Adults: A Cohort Study in the US SEER Cancer Registries. Lancet Oncol (2011) 12:353–60. 10.1016/S1470-2045(11)70061-4 PMC3086738 21454129

[5] Donin N Filson C Drakaki A Tan HJ Castillo A Kwan L Risk of second primary malignancies among cancer survivors in the United States, 1992 through 2008. Cancer (2016) 122:3075–86. 10.1002/cncr.30164 PMC6192520 27377470

[6] Lönn S Gilbert ES Ron E Smith SA Stovall M Curtis RE Comparison of second cancer risks from brachytherapy and external beam therapy after uterine corpus cancer. Cancer Epidemiol Biomarkers Prev (2010) 19:464–74. 10.1158/1055-9965.EPI-09-0892 PMC2866968 20142245

[7] Abdel-Wahab M Reis IM Hamilton K Second primary cancer after radiotherapy for prostate cancer–a seer analysis of brachytherapy versus external beam radiotherapy. Int J Radiat Oncol Biol Phys (2008) 72:58–68. 10.1016/j.ijrobp.2007.12.043 18374503

[8] Birgisson H Påhlman L Gunnarsson U Glimelius B Occurrence of second cancers in patients treated with radiotherapy for rectal cancer. J Clin Oncol (2005) 23:6126–31. 10.1200/JCO.2005.02.543 16135478

[9] Huo D Hetzel JT Roy H Rubin DT Association of colorectal cancer and prostate cancer and impact of radiation therapy. Cancer Epidemiol Biomarkers Prev (2009) 18:1979–85. 10.1158/1055-9965.EPI-09-0241 19531678

[10] Baxter NN Tepper JE Durham SB Rothenberger DA Virnig BA Increased risk of rectal cancer after prostate radiation: a population-based study. Gastroenterology (2005) 128:819–24. 10.1053/j.gastro.2004.12.038 15825064

[11] Majewski W Majewski S Maciejewski A Kolosza Z Tarnawski R Adverse Effects After Radiotherapy for Early Stage (I,IIa,IIb) Seminoma. Radiother Oncol (2005) 76:257–63. 10.1016/j.radonc.2005.04.003 15921773

[12] Rombouts AJM Hugen N Elferink MAG Poortmans PMP Nagtegaal ID de Wilt JHW Increased Risk for Second Primary Rectal Cancer After Pelvic Radiation Therapy. Eur J Cancer (2020) 124:142–51. 10.1016/j.ejca.2019.10.022 31765989

[13] Hinnen KA Schaapveld M van Vulpen M Battermann JJ van der Poel H van Oort IM Prostate Brachytherapy and Second Primary Cancer Risk: A Competitive Risk Analysis. J Clin Oncol (2011) 29:4510–5. 10.1200/JCO.2011.35.0991 22025166

[14] Wiltink LM Nout RA Fiocco M Kranenbarg EM-K Jürgenliemk-Schulz IM Jobsen JJ No Increased Risk of Second Cancer After Radiotherapy in Patients Treated for Rectal or Endometrial Cancer in the Randomized TME, PORTEC-1, and PORTEC-2 Trials. J Clin Oncol (2015) 33:1640–6. 10.1200/JCO.2014.58.6693 25534376

[15] Murray LJ Thompson CM Lilley J Cosgrove V Franks K Sebag-Montefiore D Radiation-induced Second Primary Cancer Risks From Modern External Beam Radiotherapy for Early Prostate Cancer: Impact of Stereotactic Ablative Radiotherapy (SABR), Volumetric Modulated Arc Therapy (VMAT) and Flattening Filter Free (FFF) Radiotherapy. Phys Med Biol (2015) 60:1237–57. 10.1088/0031-9155/60/3/1237 25590229

[16] Rombouts AJM Hugen N van Beek JJP Poortmans PMP de Wilt JHW Nagtegaal ID Does Pelvic Radiation Increase Rectal Cancer Incidence? - A Systematic Review and Meta-Analysis. Cancer Treat Rev (2018) 68:136–44. 10.1016/j.ctrv.2018.05.008 29957373

[17] Werbrouck J Ost P Fonteyne V De Meerleer G De Neve W Bogaert E Early biomarkers related to secondary primary cancer risk in radiotherapy treated prostate cancer patients: IMRT versus IMAT. Radiother Oncol (2013) 107:377–81. 10.1016/j.radonc.2013.05.014 23791364

[18] van der Meij W Rombouts AJM Rütten H Bremers AJA de Wilt JHW Treatment of Locally Recurrent Rectal Carcinoma in Previously (Chemo)Irradiated Patients: A Review. Dis Colon Rectum (2016) 59:148–56. 10.1097/DCR.0000000000000547 26734974

[19] Valentini V Morganti AG Gambacorta MA Mohiuddin M Doglietto GB Coco C Preoperative Hyperfractionated Chemoradiation for Locally Recurrent Rectal Cancer in Patients Previously Irradiated to the Pelvis: A Multicentric Phase II Study. Int J Radiat Oncol Biol Phys (2006) 64:1129–39. 10.1016/j.ijrobp.2005.09.017 16414206

[20] Margel D Baniel J Wasserberg N Bar-Chana M Yossepowitch O Radiation therapy for prostate cancer increases the risk of subsequent rectal cancer. Ann Surg (2011) 254:947–50. 10.1097/SLA.0b013e3182382fd5 22107741

[21] Preston DL Ron E Tokuoka S Funamoto S Nishi N Soda M Solid cancer incidence in atomic bomb survivors: 1958-1998. Radiat Res (2007) 168:1–64. 10.1667/RR0763.1 17722996

[22] de Gonzalez AB Wong J Kleinerman R Kim C Morton L Bekelman JE Risk of Second Cancers According to Radiation Therapy Technique and Modality in Prostate Cancer Survivors. Int J Radiat Oncol Biol Phys (2015) 91:295–302. 10.1016/j.ijrobp.2014.10.040 25636756 PMC4484296

[23] Sud A Thomsen H Sundquist K Houlston RS Hemminki K Risk of Second Cancer in Hodgkin Lymphoma Survivors and Influence of Family History. J Clin Oncol (2017) 35:1584–90. 10.1200/JCO.2016.70.9709 PMC5455705 28384078

