# Supplementary material for: Risk and Prognosis of Secondary Rectal Cancer After Radiation Therapy for Pelvic Cancer
Source: Front Oncol. 2020 Oct 29;10:584072. doi: 10.3389/fonc.2020.584072 (PMC7658675; doi:10.3389/fonc.2020.584072)
Supplement: Supplementary file 1 [file Table_1.docx]

Supplementary Material

# Supplementary Tables

## Table S1, NRT-SPRC and OPRC patients.

|  | NRT-SPRC n=866 | OPRC n=94394 | p value |
| --- | --- | --- | --- |
|  | n (%) or  Mean ± SD | n (%) or  Mean ± SD |  |
| Gender |  |  | <0.001 |
| Male | 558 (64.4) | 53091 (56.2) |  |
| Female | 308 (35.6) | 41303 (43.8) |  |
| Race |  |  | 0.024 |
| White | 743 (85.8) | 77771 (82.4) |  |
| Black | 58 ( 6.7) | 7386 ( 7.8) |  |
| Other | 65 ( 7.5) | 9237 ( 9.8) |  |
| Unknown | 0 ( 0.0) | 406 ( 0.4) |  |
| Age at diagnosis | 74.49 ± 10.27 | 65.03 ± 13.31 | <0.001 |
| Radiotherapy |  |  | 0.105 |
| Yes | 265 (30.6) | 31399 (33.3) |  |
| No | 601 (69.4) | 62995 (66.7) |  |
| Chemotherapy |  |  | 0.358 |
| Yes | 287 (33.1) | 32747 (34.7) |  |
| No |  |  |  |
| Surgery |  |  | <0.001 |
| Yes | 679 (78.4) | 36952 (39.1) |  |
| No | 187 (21.6) | 57442 (60.9) |  |
| Stage |  |  | <0.001 |
| Localized | 440 (50.8) | 40808 (43.2) |  |
| Regional | 289 (33.4) | 35391 (37.5) |  |
| Distant | 137 (15.8) | 18195 (19.3) |  |

NRT-SPRC, SPRC without RT

OPRC, only primary rectal cancer

## Table S2, Matched NRT-SPRC and OPRC patients.

|  | NRT-SPRC n=863 | OPRC n=4300 | p value |
| --- | --- | --- | --- |
|  | n (%) or  Mean ± SD | n (%) or  Mean ± SD |  |
| Gender |  |  | 0.295 |
| Male | 555 (64.3) | 2848 (66.2) |  |
| Female | 308 (35.7) | 1452 (33.8) |  |
| Race |  |  | 0.9 |
| White | 741 (85.9) | 3710 (86.3) |  |
| Black | 58 (6.7) | 265 (6.2) |  |
| Other | 64 (7.4) | 325 (7.5) |  |
| Age at diagnosis | 74.42 ± 10.23 | 73.70 ± 10.81 | 0.071 |
| Radiotherapy |  |  | 0.473 |
| Yes | 264 (30.6) | 1372 (31.9) |  |
| No | 599 (69.4) | 2928 (68.1) |  |
| Chemotherapy |  |  | 0.528 |
| Yes | 286 (33.1) | 1476 (34.3) |  |
| No | 577 (66.9) | 2824 (65.7) |  |
| Surgery |  |  | 0.22 |
| Yes | 676 (78.3) | 3450 (80.2) |  |
| No | 187 (21.6) | 850 (19.8) |  |
| Stage |  |  | 0.879 |
| Localized | 439 (50.9) | 2212 (51.4) |  |
| Regional | 288 (33.4) | 1439 (33.5) |  |
| Distant | 136 (15.8) | 649 (15.1) |  |

NRT-SPRC, SPRC without RT

OPRC, only primary rectal cancer

## Table S3, RT-SPRC and OPRC patients.

|  | RT-SPRC n=428 | OPRC n=94394 | p value |
| --- | --- | --- | --- |
|  | n (%) or  Mean ± SD | n (%) or  Mean ± SD |  |
| Gender |  |  | 0.021 |
| Male | 265 (61.9) | 53091 (56.2) |  |
| Female | 163 (39.1) | 41303 (43.8) |  |
| Race |  |  | 0.159 |
| White | 350 (81.8) | 77771 (82.4) |  |
| Black | 43 (10.0) | 7386 ( 7.8) |  |
| Other | 35 ( 8.2) | 9237 ( 9.8) |  |
| Age at diagnosis | 75.46 ± 9.19 | 65.03 ± 13.31 | <0.001 |
| Radiotherapy |  |  | <0.001 |
| Yes | 45 (10.5) | 31399 (33.3) |  |
| No | 383 (89.5) | 62995 (66.7) |  |
| Chemotherapy |  |  | <0.001 |
| Yes | 113 (26.4) | 32747 (34.7) |  |
| No | 315 (73.6) | 61647 (65.3) |  |
| Surgery |  |  | <0.001 |
| Yes | 341 (79.7) | 36952 (39.1) |  |
| No | 87 (20.3) | 57442 (60.9) |  |
| Stage |  |  | 0.033 |
| Localized | 207 (48.4) | 40808 (43.2) |  |
| Regional | 157 (36.7) | 35391 (37.5) |  |
| Distant | 64 (15.0) | 18195 (19.3) |  |

RT-SPRC, SPRC with RT

OPRC, only primary rectal cancer

## Table S4, Matched RT-SPRC and OPRC patients.

|  | RT-SPRC n=428 | OPRC n=2110 | p value |
| --- | --- | --- | --- |
|  | n (%) or  Mean ± SD | n (%) or  Mean ± SD |  |
| Gender |  |  | 0.002 |
| Male | 265 (61.9) | 1468 (69.6) |  |
| Female | 163 (38.1) | 642 (30.4) |  |
| Race |  |  | 0.52 |
| White | 350 (81.8) | 1727 (81.8) |  |
| Black | 43 (10.0) | 184 ( 8.7) |  |
| Other | 35 ( 8.2) | 199 ( 9.4) |  |
| Age at diagnosis | 75.46 ± 9.19 | 74.48 ± 10.77 | 0.077 |
| Radiotherapy |  |  | 0.837 |
| Yes | 45 (10.5) | 232 (10.0) |  |
| No | 383 (89.5) | 1878 (89.0) |  |
| Chemotherapy |  |  | 0.748 |
| Yes | 315 (73.6) | 1534 (72.7) |  |
| No | 113 (26.4) | 576 (27.3) |  |
| Surgery |  |  | 0.62 |
| Yes | 341 (79.7) | 1706 (80.9) |  |
| No | 87 (20.3) | 404 (19.1) |  |
| Stage |  |  | 0.942 |
| Localized | 207 (48.4) | 1028 (48.7) |  |
| Regional | 157 (36.7) | 757 (35.9) |  |
| Distant | 64 (15.0) | 325 (15.4) |  |

RT-SPRC, SPRC with RT

OPRC, only primary rectal cancer
